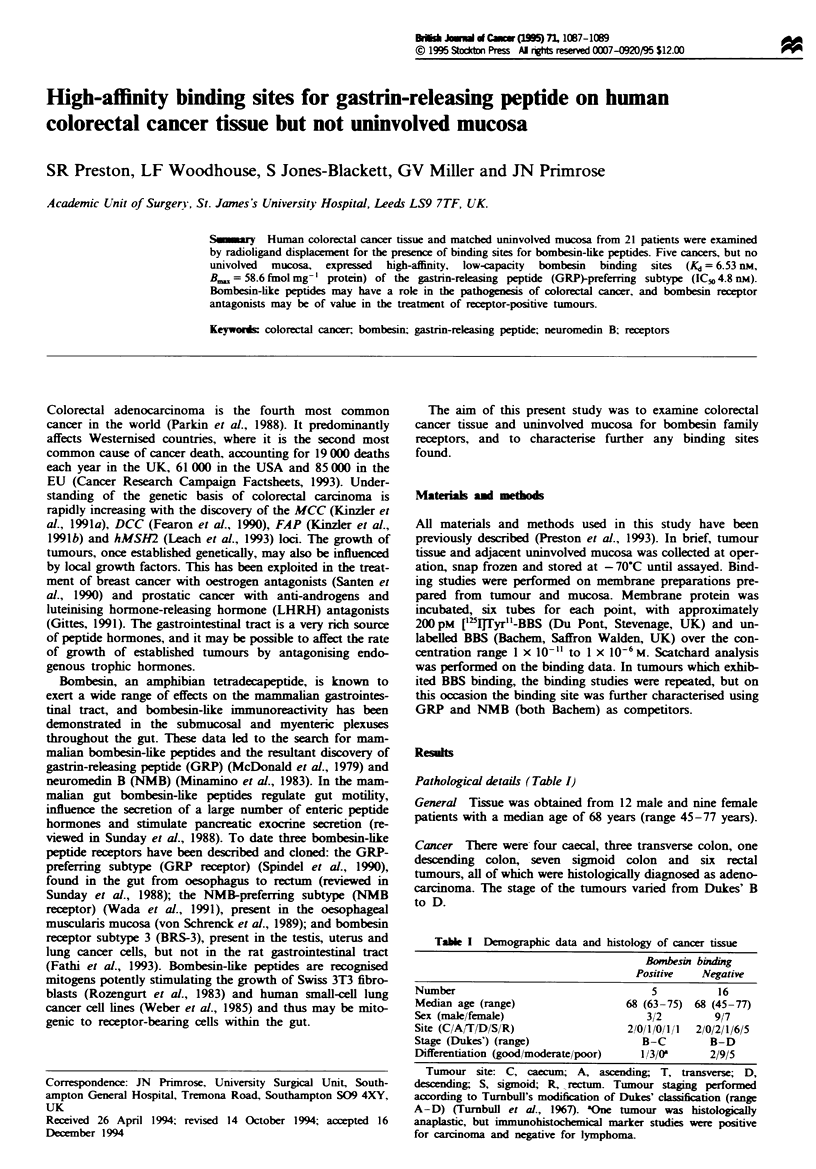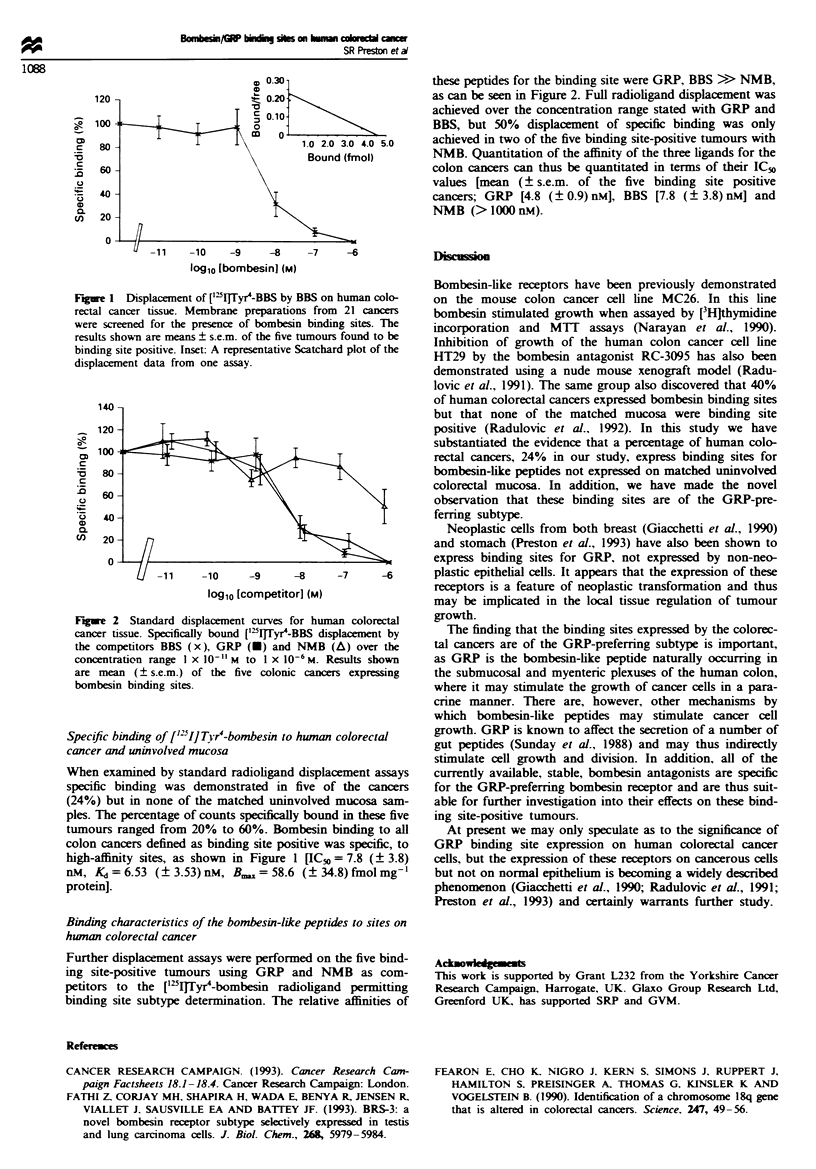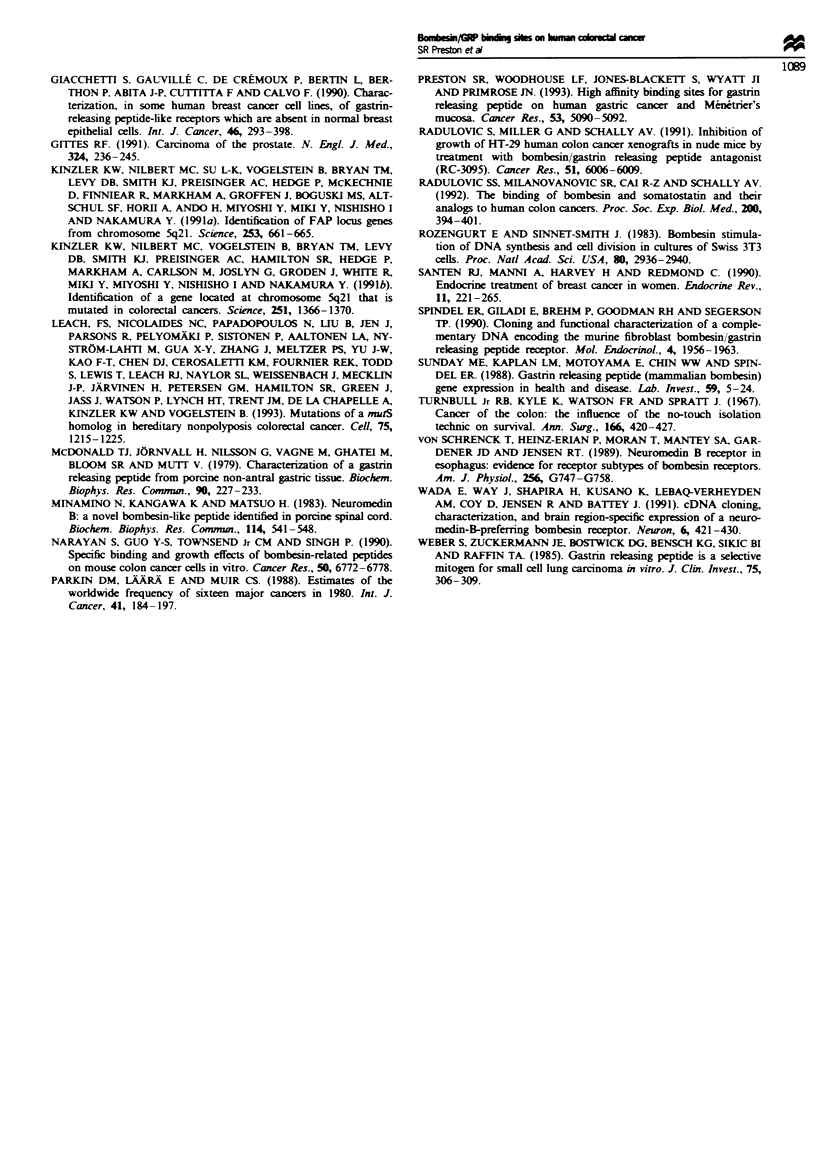# High-affinity binding sites for gastrin-releasing peptide on human colorectal cancer tissue but not uninvolved mucosa.

**DOI:** 10.1038/bjc.1995.210

**Published:** 1995-05

**Authors:** S. R. Preston, L. F. Woodhouse, S. Jones-Blackett, G. V. Miller, J. N. Primrose

**Affiliations:** Academic Unit of Surgery, St. James's University Hospital, Leeds, UK.

## Abstract

Human colorectal cancer tissue and matched uninvolved mucosa from 21 patients were examined by radioligand displacement for the presence of binding sites for bombesin-like peptides. Five cancers, but no uninvolved mucosa, expressed high-affinity, low-capacity bombesin binding sites (Kd = 6.53 nM, Bmax = 58.6 fmol mg-1 protein) of the gastrin-releasing peptide (GRP)-preferring subtype (IC50 4.8 nM). Bombesin-like peptides may have a role in the pathogenesis of colorectal cancer, and bombesin receptor antagonists may be of value in the treatment of receptor-positive tumours.


British Journal of Cancer (1995) 71, 1087-1089

© 1995 Stockton Press All rights reserved 0007-0920/95 $12.00

### High-affinity binding sites for gastrin-releasing peptide on human colorectal cancer tissue but not uninvolved mucosa

SR Preston, LF Woodhouse, S Jones-Blackett, GV Miller and JN Primrose

*Academic Unit of Surgery, St. James's University Hospital, Leeds LS9 7TF, UK.*

**Summary** Human colorectal cancer tissue and matched uninvolved mucosa from 21 patients were examined by radioligand displacement for the presence of binding sites for bombesin-like peptides. Five cancers, but no univolved mucosa, expressed high-affinity, low-capacity bombesin binding sites ($K_d$ = 6.53 nM, $B_{max}$ = 58.6 fmol $mg^{-1}$ protein) of the gastrin-releasing peptide (GRP)-preferring subtype ($IC_{50}$ 4.8 nM). Bombesin-like peptides may have a role in the pathogenesis of colorectal cancer, and bombesin receptor antagonists may be of value in the treatment of receptor-positive tumours.

**Keywords:** colorectal cancer; bombesin; gastrin-releasing peptide; neuromedin B; receptors

Colorectal adenocarcinoma is the fourth most common cancer in the world (Parkin *et al.*, 1988). It predominantly affects Westernised countries, where it is the second most common cause of cancer death, accounting for 19 000 deaths each year in the UK, 61 000 in the USA and 85 000 in the EU (Cancer Research Campaign Factsheets, 1993). Understanding of the genetic basis of colorectal carcinoma is rapidly increasing with the discovery of the *MCC* (Kinzler *et al.*, 1991*a*), *DCC* (Fearon *et al.*, 1990), *FAP* (Kinzler *et al.*, 1991*b*) and *hMSH2* (Leach *et al.*, 1993) loci. The growth of tumours, once established genetically, may also be influenced by local growth factors. This has been exploited in the treatment of breast cancer with oestrogen antagonists (Santen *et al.*, 1990) and prostatic cancer with anti-androgens and luteinising hormone-releasing hormone (LHRH) antagonists (Gittes, 1991). The gastrointestinal tract is a very rich source of peptide hormones, and it may be possible to affect the rate of growth of established tumours by antagonising endogenous trophic hormones.

Bombesin, an amphibian tetradecapeptide, is known to exert a wide range of effects on the mammalian gastrointestinal tract, and bombesin-like immunoreactivity has been demonstrated in the submucosal and myenteric plexuses throughout the gut. These data led to the search for mammalian bombesin-like peptides and the resultant discovery of gastrin-releasing peptide (GRP) (McDonald *et al.*, 1979) and neuromedin B (NMB) (Minamino *et al.*, 1983). In the mammalian gut bombesin-like peptides regulate gut motility, influence the secretion of a large number of enteric peptide hormones and stimulate pancreatic exocrine secretion (reviewed in Sunday *et al.*, 1988). To date three bombesin-like peptide receptors have been described and cloned: the GRP-preferring subtype (GRP receptor) (Spindel *et al.*, 1990), found in the gut from oesophagus to rectum (reviewed in Sunday *et al.*, 1988); the NMB-preferring subtype (NMB receptor) (Wada *et al.*, 1991), present in the oesophageal muscularis mucosa (von Schrenck *et al.*, 1989); and bombesin receptor subtype 3 (BRS-3), present in the testis, uterus and lung cancer cells, but not in the rat gastrointestinal tract (Fathi *et al.*, 1993). Bombesin-like peptides are recognised mitogens potently stimulating the growth of Swiss 3T3 fibroblasts (Rozengurt *et al.*, 1983) and human small-cell lung cancer cell lines (Weber *et al.*, 1985) and thus may be mitogenic to receptor-bearing cells within the gut.

Correspondence: JN Primrose, University Surgical Unit, Southampton General Hospital, Tremona Road, Southampton SO9 4XY, UK
Received 26 April 1994; revised 14 October 1994; accepted 16 December 1994

The aim of this present study was to examine colorectal cancer tissue and uninvolved mucosa for bombesin family receptors, and to characterise further any binding sites found.

### Materials and methods

All materials and methods used in this study have been previously described (Preston *et al.*, 1993). In brief, tumour tissue and adjacent uninvolved mucosa was collected at operation, snap frozen and stored at −70°C until assayed. Binding studies were performed on membrane preparations prepared from tumour and mucosa. Membrane protein was incubated, six tubes for each point, with approximately 200 pM $[^{125}I][Tyr^{11}]$-BBS (Du Pont, Stevenage, UK) and unlabelled BBS (Bachem, Saffron Walden, UK) over the concentration range $1 \times 10^{-11}$ to $1 \times 10^{-6}$ M. Scatchard analysis was performed on the binding data. In tumours which exhibited BBS binding, the binding studies were repeated, but on this occasion the binding site was further characterised using GRP and NMB (both Bachem) as competitors.

### Results

*Pathological details (Table I)*

*General* Tissue was obtained from 12 male and nine female patients with a median age of 68 years (range 45–77 years).

*Cancer* There were four caecal, three transverse colon, one descending colon, seven sigmoid colon and six rectal tumours, all of which were histologically diagnosed as adenocarcinoma. The stage of the tumours varied from Dukes' B to D.

**Table I** Demographic data and histology of cancer tissue

| | *Bombesin binding* | |
|---|---|---|
| | *Positive* | *Negative* |
| Number | 5 | 16 |
| Median age (range) | 68 (63–75) | 68 (45–77) |
| Sex (male/female) | 3/2 | 9/7 |
| Site (C/A/T/D/S/R) | 2/0/1/0/1/1 | 2/0/2/1/6/5 |
| Stage (Dukes') (range) | B–C | B–D |
| Differentiation (good/moderate/poor) | 1/3/0[a] | 2/9/5 |

Tumour site: C, caecum; A, ascending; T, transverse; D, descending; S, sigmoid; R, rectum. Tumour staging performed according to Turnbull's modification of Dukes' classification (range A–D) (Turnbull *et al.*, 1967). [a]One tumour was histologically anaplastic, but immunohistochemical marker studies were positive for carcinoma and negative for lymphoma.

**Figure 1** Displacement of [$^{125}$I]Tyr$^{4}$-BBS by BBS on human colorectal cancer tissue. Membrane preparations from 21 cancers were screened for the presence of bombesin binding sites. The results shown are means ± s.e.m. of the five tumours found to be binding site positive. Inset: A representative Scatchard plot of the displacement data from one assay.

**Figure 2** Standard displacement curves for human colorectal cancer tissue. Specifically bound [$^{125}$I]Tyr$^{4}$-BBS displacement by the competitors BBS (×), GRP (■) and NMB (Δ) over the concentration range $1 \times 10^{-11}$ M to $1 \times 10^{-6}$ M. Results shown are mean (± s.e.m.) of the five colonic cancers expressing bombesin binding sites.

### *Specific binding of [$^{125}$I]Tyr$^{4}$-bombesin to human colorectal cancer and uninvolved mucosa*

When examined by standard radioligand displacement assays specific binding was demonstrated in five of the cancers (24%) but in none of the matched uninvolved mucosa samples. The percentage of counts specifically bound in these five tumours ranged from 20% to 60%. Bombesin binding to all colon cancers defined as binding site positive was specific, to high-affinity sites, as shown in Figure 1 [$IC_{50}$ = 7.8 (± 3.8) nM, $K_d$ = 6.53 (± 3.53) nM, $B_{max}$ = 58.6 (± 34.8) fmol mg$^{-1}$ protein].

### *Binding characteristics of the bombesin-like peptides to sites on human colorectal cancer*

Further displacement assays were performed on the five binding site-positive tumours using GRP and NMB as competitors to the [$^{125}$I]Tyr$^{4}$-bombesin radioligand permitting binding site subtype determination. The relative affinities of these peptides for the binding site were GRP, BBS ≫ NMB, as can be seen in Figure 2. Full radioligand displacement was achieved over the concentration range stated with GRP and BBS, but 50% displacement of specific binding was only achieved in two of the five binding site-positive tumours with NMB. Quantitation of the affinity of the three ligands for the colon cancers can thus be quantitated in terms of their $IC_{50}$ values [mean (± s.e.m. of the five binding site positive cancers; GRP [4.8 (± 0.9) nM], BBS [7.8 (± 3.8) nM] and NMB (> 1000 nM).

### Discussion

Bombesin-like receptors have been previously demonstrated on the mouse colon cancer cell line MC26. In this line bombesin stimulated growth when assayed by [$^{3}$H]thymidine incorporation and MTT assays (Narayan *et al.*, 1990). Inhibition of growth of the human colon cancer cell line HT29 by the bombesin antagonist RC-3095 has also been demonstrated using a nude mouse xenograft model (Radulovic *et al.*, 1991). The same group also discovered that 40% of human colorectal cancers expressed bombesin binding sites but that none of the matched mucosa were binding site positive (Radulovic *et al.*, 1992). In this study we have substantiated the evidence that a percentage of human colorectal cancers, 24% in our study, express binding sites for bombesin-like peptides not expressed on matched uninvolved colorectal mucosa. In addition, we have made the novel observation that these binding sites are of the GRP-preferring subtype.

Neoplastic cells from both breast (Giacchetti *et al.*, 1990) and stomach (Preston *et al.*, 1993) have also been shown to express binding sites for GRP, not expressed by non-neoplastic epithelial cells. It appears that the expression of these receptors is a feature of neoplastic transformation and thus may be implicated in the local tissue regulation of tumour growth.

The finding that the binding sites expressed by the colorectal cancers are of the GRP-preferring subtype is important, as GRP is the bombesin-like peptide naturally occurring in the submucosal and myenteric plexuses of the human colon, where it may stimulate the growth of cancer cells in a paracrine manner. There are, however, other mechanisms by which bombesin-like peptides may stimulate cancer cell growth. GRP is known to affect the secretion of a number of gut peptides (Sunday *et al.*, 1988) and may thus indirectly stimulate cell growth and division. In addition, all of the currently available, stable, bombesin antagonists are specific for the GRP-preferring bombesin receptor and are thus suitable for further investigation into their effects on these binding site-positive tumours.

At present we may only speculate as to the significance of GRP binding site expression on human colorectal cancer cells, but the expression of these receptors on cancerous cells but not on normal epithelium is becoming a widely described phenomenon (Giacchetti *et al.*, 1990; Radulovic *et al.*, 1991; Preston *et al.*, 1993) and certainly warrants further study.

**Acknowledgements**

This work is supported by Grant L232 from the Yorkshire Cancer Research Campaign, Harrogate, UK. Glaxo Group Research Ltd, Greenford UK, has supported SRP and GVM.

### References

CANCER RESEARCH CAMPAIGN. (1993). *Cancer Research Campaign Factsheets 18.1–18.4*. Cancer Research Campaign: London.

FATHI Z, CORJAY MH, SHAPIRA H, WADA E, BENYA R, JENSEN R, VIALLET J, SAUSVILLE EA AND BATTEY JF. (1993). BRS-3: a novel bombesin receptor subtype selectively expressed in testis and lung carcinoma cells. *J. Biol. Chem.*, **268,** 5979–5984.

FEARON E, CHO K, NIGRO J, KERN S, SIMONS J, RUPPERT J, HAMILTON S, PREISINGER A, THOMAS G, KINSLER K AND VOGELSTEIN B. (1990). Identification of a chromosome 18q gene that is altered in colorectal cancers. *Science*, **247,** 49–56.

GIACCHETTI S, GAUVILLÉ C, DE CRÉMOUX P, BERTIN L, BERTHON P, ABITA J-P, CUTTITTA F AND CALVO F. (1990). Characterization, in some human breast cancer cell lines, of gastrin-releasing peptide-like receptors which are absent in normal breast epithelial cells. *Int. J. Cancer*, **46**, 293–398.

GITTES RF. (1991). Carcinoma of the prostate. *N. Engl. J. Med.*, **324**, 236–245.

KINZLER KW, NILBERT MC, SU L-K, VOGELSTEIN B, BRYAN TM, LEVY DB, SMITH KJ, PREISINGER AC, HEDGE P, McKECHNIE D, FINNIEAR R, MARKHAM A, GROFFEN J, BOGUSKI MS, ALTSCHUL SF, HORII A, ANDO H, MIYOSHI Y, MIKI Y, NISHISHO I AND NAKAMURA Y. (1991*a*). Identification of FAP locus genes from chromosome 5q21. *Science*, **253**, 661–665.

KINZLER KW, NILBERT MC, VOGELSTEIN B, BRYAN TM, LEVY DB, SMITH KJ, PREISINGER AC, HAMILTON SR, HEDGE P, MARKHAM A, CARLSON M, JOSLYN G, GRODEN J, WHITE R, MIKI Y, MIYOSHI Y, NISHISHO I AND NAKAMURA Y. (1991*b*). Identification of a gene located at chromosome 5q21 that is mutated in colorectal cancers. *Science*, **251**, 1366–1370.

LEACH, FS, NICOLAIDES NC, PAPADOPOULOS N, LIU B, JEN J, PARSONS R, PELYOMÄKI P, SISTONEN P, AALTONEN LA, NYSTRÖM-LAHTI M, GUA X-Y, ZHANG J, MELTZER PS, YU J-W, KAO F-T, CHEN DJ, CEROSALETTI KM, FOURNIER REK, TODD S, LEWIS T, LEACH RJ, NAYLOR SL, WEISSENBACH J, MECKLIN J-P, JÄRVINEN H, PETERSEN GM, HAMILTON SR, GREEN J, JASS J, WATSON P, LYNCH HT, TRENT JM, DE LA CHAPELLE A, KINZLER KW AND VOGELSTEIN B. (1993). Mutations of a *mutS* homolog in hereditary nonpolyposis colorectal cancer. *Cell*, **75**, 1215–1225.

McDONALD TJ, JÖRNVALL H, NILSSON G, VAGNE M, GHATEI M, BLOOM SR AND MUTT V. (1979). Characterization of a gastrin releasing peptide from porcine non-antral gastric tissue. *Biochem. Biophys. Res. Commun.*, **90**, 227–233.

MINAMINO N, KANGAWA K AND MATSUO H. (1983). Neuromedin B: a novel bombesin-like peptide identified in porcine spinal cord. *Biochem. Biophys. Res. Commun.*, **114**, 541–548.

NARAYAN S, GUO Y-S, TOWNSEND Jr CM AND SINGH P. (1990). Specific binding and growth effects of bombesin-related peptides on mouse colon cancer cells in vitro. *Cancer Res.*, **50**, 6772–6778.

PARKIN DM, LÄÄRÄ E AND MUIR CS. (1988). Estimates of the worldwide frequency of sixteen major cancers in 1980. *Int. J. Cancer*, **41**, 184–197.

PRESTON SR, WOODHOUSE LF, JONES-BLACKETT S, WYATT JI AND PRIMROSE JN. (1993). High affinity binding sites for gastrin releasing peptide on human gastric cancer and Ménétrier's mucosa. *Cancer Res.*, **53**, 5090–5092.

RADULOVIC S, MILLER G AND SCHALLY AV. (1991). Inhibition of growth of HT-29 human colon cancer xenografts in nude mice by treatment with bombesin/gastrin releasing peptide antagonist (RC-3095). *Cancer Res.*, **51**, 6006–6009.

RADULOVIC SS, MILANOVANOVIC SR, CAI R-Z AND SCHALLY AV. (1992). The binding of bombesin and somatostatin and their analogs to human colon cancers. *Proc. Soc. Exp. Biol. Med.*, **200**, 394–401.

ROZENGURT E AND SINNET-SMITH J. (1983). Bombesin stimulation of DNA synthesis and cell division in cultures of Swiss 3T3 cells. *Proc. Natl Acad. Sci. USA*, **80**, 2936–2940.

SANTEN RJ, MANNI A, HARVEY H AND REDMOND C. (1990). Endocrine treatment of breast cancer in women. *Endocrine Rev.*, **11**, 221–265.

SPINDEL ER, GILADI E, BREHM P, GOODMAN RH AND SEGERSON TP. (1990). Cloning and functional characterization of a complementary DNA encoding the murine fibroblast bombesin/gastrin releasing peptide receptor. *Mol. Endocrinol.*, **4**, 1956–1963.

SUNDAY ME, KAPLAN LM, MOTOYAMA E, CHIN WW AND SPINDEL ER. (1988). Gastrin releasing peptide (mammalian bombesin) gene expression in health and disease. *Lab. Invest.*, **59**, 5–24.

TURNBULL Jr RB, KYLE K, WATSON FR AND SPRATT J. (1967). Cancer of the colon: the influence of the no-touch isolation technic on survival. *Ann. Surg.*, **166**, 420–427.

VON SCHRENCK T, HEINZ-ERIAN P, MORAN T, MANTEY SA, GARDENER JD AND JENSEN RT. (1989). Neuromedin B receptor in esophagus: evidence for receptor subtypes of bombesin receptors. *Am. J. Physiol.*, **256**, G747–G758.

WADA E, WAY J, SHAPIRA H, KUSANO K, LEBAQ-VERHEYDEN AM, COY D, JENSEN R AND BATTEY J. (1991). cDNA cloning, characterization, and brain region-specific expression of a neuromedin-B-preferring bombesin receptor. *Neuron*, **6**, 421–430.

WEBER S, ZUCKERMANN JE, BOSTWICK DG, BENSCH KG, SIKIC BI AND RAFFIN TA. (1985). Gastrin releasing peptide is a selective mitogen for small cell lung carcinoma *in vitro*. *J. Clin. Invest.*, **75**, 306–309.